# Electronic Alerts with Automated Consultations Promote Appropriate Antimicrobial Prescriptions

**DOI:** 10.1371/journal.pone.0160551

**Published:** 2016-08-17

**Authors:** Moonsuk Kim, Kyoung-Ho Song, Chung-Jong Kim, Minkyo Song, Pyoeng Gyun Choe, Wan Beom Park, Ji Hwan Bang, Hee Hwang, Eu Suk Kim, Sang-Won Park, Nam Joong Kim, Myoung-don Oh, Hong Bin Kim

**Affiliations:** 1 Department of Internal Medicine, Seoul National University Bundang Hospital, Seongnam, Republic of Korea; 2 Department of Internal Medicine, Seoul National University College of Medicine, Seoul, Republic of Korea; 3 Department of Preventive Medicine, Seoul National University College of Medicine, Seoul, Republic of Korea; 4 Center of Medical Informatics, Seoul National University Bundang Hospital, Seongnam, Republic of Korea; Azienda Ospedaliera Universitaria di Perugia, ITALY

## Abstract

**Background:**

To promote appropriate antimicrobial use in bloodstream infections (BSIs), we initiated an intervention program consisting of electronic alerts and automated infectious diseases consultations in which the identification and antimicrobial susceptibility test (ID/AST) results were reported.

**Methods:**

We compared the appropriateness of antimicrobial prescriptions and clinical outcomes in BSIs before and after initiation of the program. Appropriateness was assessed in terms of effective therapy, optimal therapy, de-escalation therapy, and intravenous to oral switch therapy.

**Results:**

There were 648 BSI episodes in the pre-program period and 678 in the program period. The proportion of effective, optimal, and de-escalation therapies assessed 24 hours after the reporting of the ID/AST results increased from 87.8% (95% confidence interval [CI] 85.5–90.5), 64.4% (95% CI 60.8–68.1), and 10.0% (95% CI 7.5–12.6) in the pre-program period, respectively, to 94.4% (95% CI 92.7–96.1), 81.4% (95% CI 78.4–84.3), and 18.6% (95% CI 15.3–21.9) in the program period, respectively. Kaplan-Meier analyses and log-rank tests revealed that the time to effective (p<0.001), optimal (p<0.001), and de-escalation (p = 0.017) therapies were significantly different in the two periods. Segmented linear regression analysis showed the increase in the proportion of effective (p = 0.015), optimal (p<0.001), and de-escalation (p = 0.010) therapies at 24 hours after reporting, immediately after program initiation. No significant baseline trends or changes in trends were identified. There were no significant differences in time to intravenous to oral switch therapy, length of stay, and 30-day mortality rate.

**Conclusion:**

This novel form of stewardship program based on intervention by infectious disease specialists and information technology improved antimicrobial prescriptions in BSIs.

## Introduction

In the treatment of bloodstream infections (BSIs), administration of appropriate antimicrobials without delay is crucial for improving clinical outcomes [1–3]. However, in previous studies, 23% to 46% of empirical antimicrobials for BSIs were considered inappropriate [3–6]. Even after the identification and antimicrobial susceptibility test (ID/AST) results were reported, 8% to 19% of the antimicrobials prescribed remained inappropriate [5–7].

There have been several trials of interventions to improve the appropriateness of antimicrobial therapy in BSIs, some of which led to improvements in antimicrobial prescribing and clinical outcomes [6,8,9]. However, many of these interventions were highly labor-intensive. In planning sustainable interventions, the available resources, circumstances of individual hospitals, and strategies to increase efficiency should be considered.

To promote appropriate antimicrobial use in BSIs, we initiated an intervention program in August 2011 which consisted of electronic alerts and automated infectious diseases consultations, and evaluated the impact of the program on the appropriateness of antimicrobial prescriptions.

## Materials and Methods

### Hospital setting and program design

**Fig 1:**
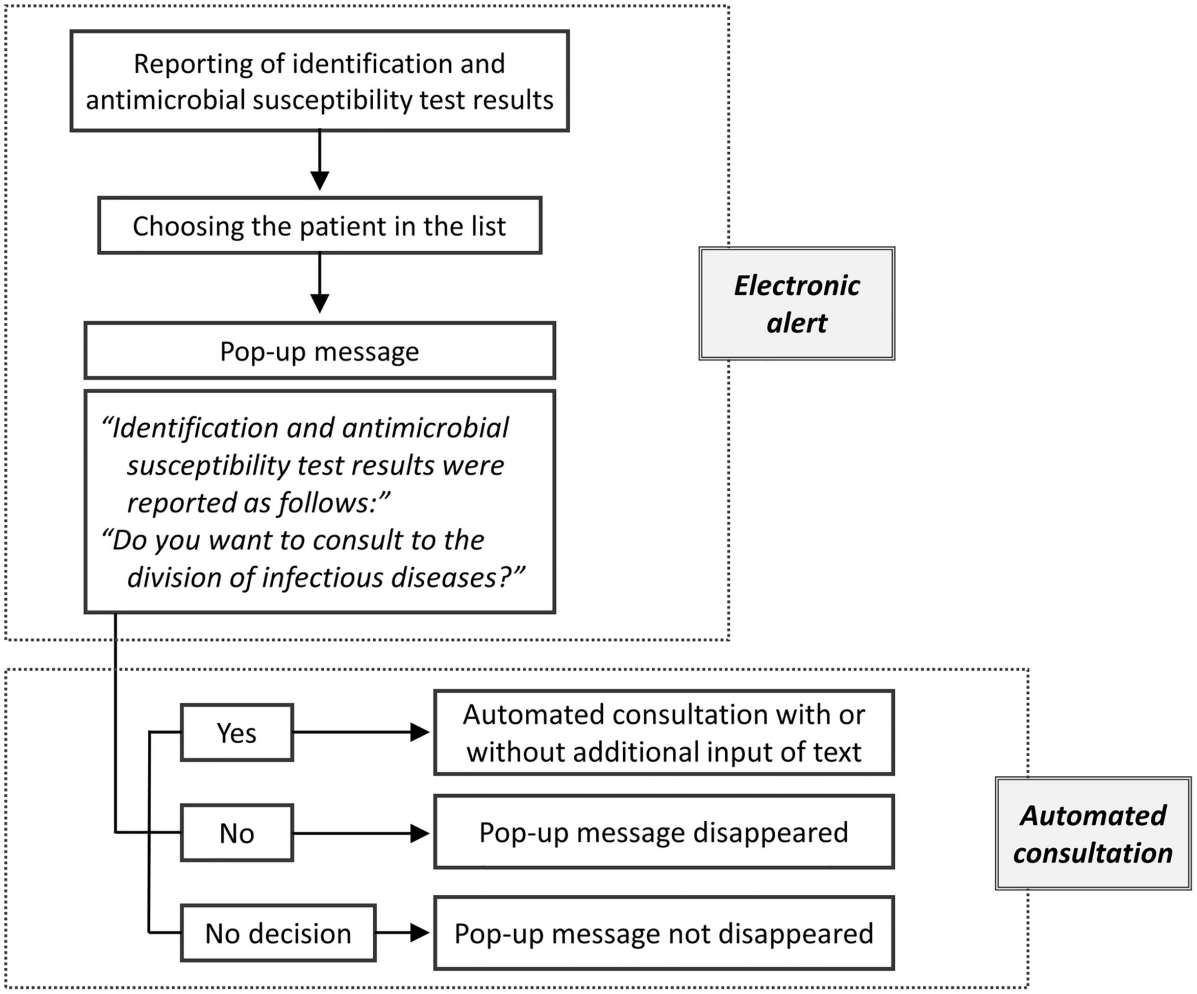
Flow diagram of electronic alerts and automated consultations.

Our institution is a tertiary teaching hospital with 900 beds. We planned an intervention program taking advantage of our health information system for electronic medical records (EMR) in our hospital. The key feature of this program is a pop-up message, which gives the ID/AST results with a note about whether a consultation with an infectious disease specialist (IDS) is needed. Attending physicians can issue the consultation according to the automated process. The only action needed to make a consultation is to click on the message. However, the consultation is not mandatory and can be canceled (Fig 1). Pop-up messages are generated for all categories of ID/AST results. Before the initiation of this intervention program, no additional process of reporting was available, and attending physicians had to open the test results section of the EMR to view the results. This program was initiated on August 19, 2011.

### Study period

We designated the period before the initiation of the program as the “pre-program period”, and the period of the program as the “program period”. Excluding the month of initiation of the program (August 2011), the total duration of the study was 2 years, comprising a 1-year pre-program period (from August 2010 to July 2011) and a 1-year program period (from September 2011 to August 2012).

### Study subjects

During the study period, all episodes of BSI in adult (≥18 years) patients were reviewed. A BSI episode was defined as growth of pathogenic bacteria or fungi from one or more sets of blood culture samples in a patient with systemic inflammatory response syndrome. When present, common skin commensals, such as *Corynebacterium* species or coagulase-negative staphylococci were considered pathogens only when two or more sets of blood cultures were positive within 48 hours, and when clinically related infection existed.

BSI episodes were included in the analysis only when blood cultures were performed for hospitalized patients or those in the emergency department. Because the program started to operate after the reporting of the ID/AST results, the episodes involving patients discharged from the hospital before the reporting were excluded. We considered that the impact of the program on antimicrobial prescription and clinical outcomes could not be assessed appropriately in cases involving patients who died within 24 hours after the reporting and patients with polymicrobial infection, defined as the isolation of two or more pathogens from the same blood culture set. Furthermore, the appropriateness of the use of antimicrobial agents for polymicrobial infections was not estimated solely on the basis of the ID/AST results. Therefore, the episodes that involved early death and polymicrobial infections were excluded from the study.

BSI onset was defined as the time when the initial blood culture was ordered. If there was a new positive culture two weeks after BSI onset, that episode was considered a separate one. However, if an obviously different infection developed, it was counted as a separate episode even when it developed within two weeks of the initial episode.

### Outcomes

Primary outcomes were the time to appropriate antimicrobial administration and the frequency of appropriate antimicrobial administration after the ID/AST results were reported. The appropriateness of antimicrobial use was assessed in terms of effective therapy, optimal therapy, de-escalation therapy, and intravenous to oral switch. Secondary outcomes were length of stay, and all-cause and infection-related mortalities 30 days from BSI onset. Infection-related mortality was defined according to the following criteria, adapted from Harbarth [10], (1) positive blood culture at the time of death, (2) a persistent focus of BSI with associated clinical signs of sepsis, and (3) death within two weeks of the documentation of BSI without an alternative explanation.

### Definitions of variables

1. Reference time: the time when the ID/AST results were reported (S1 Fig).
2. Type of acquisition: (1) community-onset BSI: BSI that occurred <48 hours after admission (or emergency department visit, if the patient was admitted via the emergency department); (2) healthcare-associated BSI: BSI that occurred <48 hours after admission with risk of healthcare exposure [11]; (3) hospital-onset BSI: BSI that occurred ≥48 hours after admission.
3. Empirical therapy: antimicrobial in use at the reference time. Antimicrobials were classified into ineffective, effective and optimal, and effective but non-optimal. Superfluous broad-spectrum antimicrobial therapy was considered effective but non-optimal.
4. Effective therapy: Effective therapy was defined as the administration of an active antimicrobial for a confirmed pathogen for each ID/AST result. Time to effective therapy was defined as the interval from the reference time to effective therapy.
5. Superfluous broad-spectrum antimicrobials: if the antimicrobials of interest were used as empirical therapy and the ID/AST results revealed that their spectra were excessive, we classified them as superfluous. For gram-negative BSI, the antimicrobials of interest were piperacillin/tazobactam, ceftazidime, cefepime, carbapenem, and polymyxin E. Glycopeptide and oxazolidinone were the antimicrobials of interest for gram-positive BSI (daptomycin and anti-MRSA cephalosporins were not available during the study period).
6. Optimal therapy: Optimal therapy was defined as the administration of an effective antimicrobial which was not one of the superfluous broad-spectrum antimicrobials and adequate in relation to the site of infection, route of administration, and dosing. Likewise, time to optimal therapy was defined as the interval from the reference time to optimal therapy.
7. De-escalation therapy: De-escalation therapy was defined as the discontinuation of use of a broad-spectrum antimicrobial and the administration of an antimicrobial with a narrower spectrum. We assumed the time of discontinuation of an antimicrobial to be the time of the next scheduled administration of the antimicrobial. The antimicrobial with a narrower spectrum was not necessarily the one with the narrowest spectrum of those available.
8. Intravenous to oral switch: Only the cases involving the switch to antimicrobials that were active in vitro were included in the analysis. We considered the switch as appropriate in cases of (1) absence of clinical indication for intravenous therapy, such as CNS infection and endocarditis, (2) normalization of body temperature, (3) decrease in the level of inflammatory markers, (4) improvement in the patient’s symptoms and signs, and (5) normal gastrointestinal absorption (records of normal oral intake without vomiting and diarrhea) [6,12].

### Statistical analysis

To compare general characteristics between the two periods, the chi-square test or Fisher's exact test was used for categorical variables, and Student’s *t*-test or the Mann-Whitney median test for continuous variables. Kaplan-Meier analysis and log-rank tests were performed to compare the time to effective therapy, optimal therapy, de-escalation therapy, and intravenous to oral switch. All data were censored at 10 days (240 hours) from the reference time. We considered the data as censored when a patient died within 10 days after reporting, or if the patient was discharged from the hospital within 10 days and was not monitored after this period.

Segmented linear regression analyses were performed to evaluate the trends of outcomes over time before and after the initiation of the intervention program [13]. Analyses were conducted using monthly data of the proportion of patients on appropriate therapy at 24 and 48 hours after the reporting of the ID/AST results. The Durbin-Watson statistic was used to detect the presence of autocorrelation.

All analyses were performed using SAS software version 9.4 (SAS Institute Inc.). All tests were two-tailed and adopted an alpha level of 0.05.

### Ethics statement

The study was approved by the Institutional Review Board of Seoul National University Bundang Hospital (IRB No. B-1412/278-113). The study was conducted with a waiver of informed consent from individual patients. Patient data were de-identified and anonymized before analysis.

## Results

A total of 879 episodes in the pre-program period and 899 episodes in the program period were identified as BSIs of clinical significance. After that, 231 episodes in the pre-program period and 221 episodes in the program period were excluded according to the exclusion criteria. Therefore, 648 episodes in the pre-program period and 678 in the program period were included in the analysis (S2 Fig). The corresponding number of patient was 566 and 591, respectively.

The time from BSI onset to the reporting of the ID/AST results was shortened in the program period by 9.0 hours (95% confidence interval [CI] 5.0–12.9 hours, p<0.001). The automated consultations were canceled by the attending physicians in 22.0% of all episodes in the program period (149 of 678). In the remaining 529 episodes, in which the consultations were not canceled, the mean interval from automated consultation forwarding to signed reply was 6.8 hours (95% CI 5.8–7.8).

### Clinical characteristics of bloodstream infection episodes

The clinical characteristics of the episodes in the two periods are compared in Table 1. There were no significant differences in median age, median age-adjusted Charlson comorbidity index [14], sex distribution, comorbidity, service department in charge, type of acquisition, primary site of infection, and classification of empirical therapy (S3 Fig). However, the proportion of ICU episodes was larger in the program period (7.1% versus 11.7%, p = 0.005).

**Table 1:** Clinical characteristics of bloodstream infection episodes in the pre-program and program periods.

	Pre-program period		Program period		*p*-value
	(n = 648)		(n = 678)		
	N	(%)	N	(%)	
Age, years					
≥65	402	(62.0)	406	(59.9)	0.421
Median (IQR)	69 (57–76)		68 (57–75)		0.521
Gender					0.779
Female	285	(44.0)	293	(43.2)	
Male	363	(56.0)	385	(56.8)	
Comorbidity					
Solid tumor	277	(42.8)	288	(42.5)	0.921
Metastatic cancer	97	(15.0)	92	(13.6)	0.466
Hematologic malignancy	49	(7.6)	57	(8.4)	0.571
Diabetes	135	(20.8)	153	(22.6)	0.444
Cerebrovascular disease	119	(18.4)	118	(17.4)	0.648
Chronic pulmonary disease	36	(5.6)	47	(6.9)	0.301
Heart failure	32	(4.9)	48	(7.1)	0.102
ESRD	28	(4.3)	36	(5.3)	0.401
Liver cirrhosis	22	(3.4)	15	(2.2)	0.191
Charlson comorbidity index					
≥5	315	(48.6)	330	(48.7)	0.982
Median (IQR)	5 (3–8)		5 (3–7)		0.475
Service department					0.493
Medical	411	(63.4)	441	(65.0)	
Surgical	159	(24.5)	145	(21.4)	
Infectious diseases	72	(11.1)	83	(12.2)	
Other	6	(0.9)	9	(1.3)	
Patient location					0.005
ICU	46	(7.1)	79	(11.7)	
Non-ICU	602	(92.9)	599	(88.4)	
Type of acquisition					0.682
Community-acquired	189	(29.2)	191	(28.2)	
Healthcare-associated	193	(29.8)	217	(32.0)	
Hospital-onset	266	(41.1)	270	(39.8)	
Site of infection					0.579
Intra-abdominal	115	(17.8)	145	(21.4)	
Hepatobiliary	195	(30.1)	181	(26.7)	
Genitourinary	135	(20.8)	127	(18.7)	
Catheter-related	59	(9.1)	82	(12.1)	
Lower respiratory	37	(5.7)	31	(4.6)	
Skin and soft tissue^a^	40	(6.2)	40	(5.9)	
Bone & joint	33	(5.1)	30	(4.4)	
Cardiovascular	18	(2.8)	19	(2.8)	
Other	16	(2.7)	23	(3.4)	
Empirical therapy					0.706
Effective	519	(80.1)	527	(77.7)	0.292
Superfluous broad-spectrum	181	(27.9)	170	(25.1)	0.238
Optimal	317	(48.9)	337	(49.4)	0.775
Other	21	(3.2)	20	(3.0)	0.760
Ineffective	129	(19.9)	151	(22.3)	0.292

^a^. Including surgical wound infection.

There were also no significant differences in the distribution of pathogens between the two periods except for cases of coagulase-negative staphylococci (2.5% versus 5.0%, p = 0.015) and *Escherichia coli* (37.8% versus 31.7%, p = 0.020). The proportion of extended-spectrum beta-lactamase (ESBL) producers among the isolates of *E*. *coli* was larger in the program period (32.2% versus 44.2%, p = 0.008). The pathogen distribution is shown in Table 2.

**Table 2:** Pathogen distribution in bloodstream infection episodes.

	Pre-program period (n = 648)		Program period (n = 678)		*p*-value
	N	(%)	N	(%)	
Gram-positive bacteria	191	(29.5)	207	(30.5)	0.675
*Staphylococcus aureus*	90	(13.9)	91	(13.4)	0.804
Methicillin-resistant	47	(52.2)	47	(51.6)	0.938
Coagulase-negative staphylococci	16	(2.5)	34	(5.0)	0.015
*Streptococcus* species	46	(7.1)	38	(5.6)	0.264
*Enterococcus* species	37	(5.7)	41	(6.1)	0.794
Other	2	(0.3)	3	(0.4)	1.000
Gram-negative bacteria	415	(64.0)	433	(63.9)	0.946
*Escherichia coli*	245	(37.8)	215	(31.7)	0.020
ESBL-producing	79	(32.2)	95	(44.2)	0.008
*Klebsiella* species	89	(13.7)	116	(17.1)	0.089
ESBL-producing	22	(24.7)	30	(25.9)	0.852
*Pseudomonas aeruginosa*	25	(3.9)	38	(5.6)	0.135
Carbapenem-resistant	5	(20.0)	5	(13.2)	0.500
*Enterobacter* species	21	(3.2)	15	(2.2)	0.249
*Proteus* species	7	(1.1)	4	(0.6)	0.376
*Ctirobacter* species	5	(0.8)	11	(1.6)	0.209
*Serratia marcescens*	4	(0.6)	6	(0.9)	0.754
*Acinetobacter* species	5	(0.8)	8	(1.2)	0.580
Other	14	(2.2)	20	(2.9)	0.363
Anaerobe	16	(2.5)	19	(2.8)	0.705
*Bacteroides* species	9	(1.4)	15	(2.2)	0.261
Other	7	(1.1)	4	(0.6)	0.376
Yeast	26	(4.0)	19	(2.8)	0.224
*Candida albicans*	8	(1.2)	6	(0.9)	0.534
Other	18	(2.8)	13	(1.9)	0.300

### Time to effective therapy

**Fig 2:**
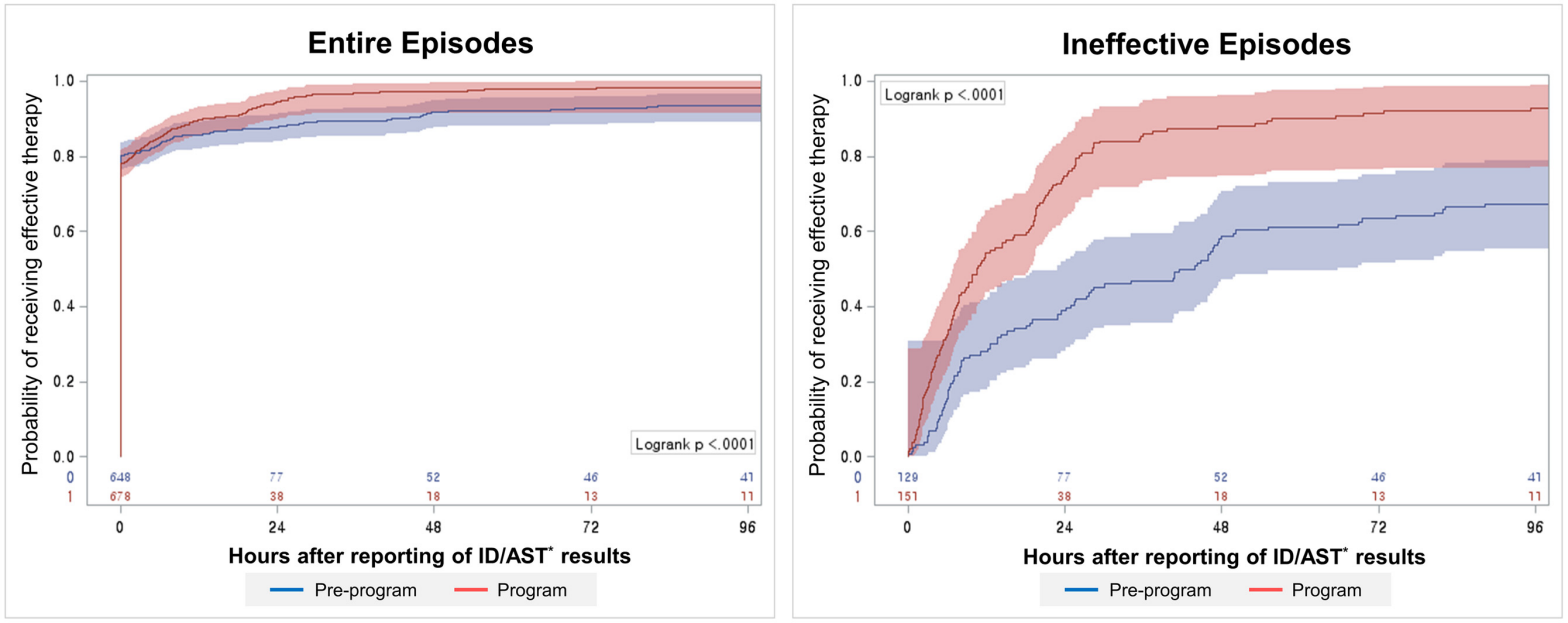
Kaplan-Meier curve of the time to effective therapy. *Identification and Antimicrobial susceptibility test.

We compared the time to effective therapy between the two periods in two steps according to the target group using Kaplan-Meier methods as shown in Fig 2. First, the analysis was performed for all episodes. The proportion of effective therapy at 24 and 48 hours after the reporting of the ID/AST results increased from 87.8% (95% CI 85.5–90.5) and 91.8% (95% CI 89.7–93.9) in the pre-program period to 94.4% (95% CI 92.7–96.1) and 97.4% (95% CI 96.1–98.6) in the program period. Subsequently, the analysis was performed only for the episodes for which empirical treatment was classified as ineffective. The proportion of effective therapy at 24 and 48 hours increased from 38.8% (95% CI 30.4–47.2) and 58.7% (95% CI 50.1–67.2) to 74.8% (95% CI 67.9–81.7) and 88.1% (95% CI 82.9–93.3). The median time to effective therapy was shortened in the program period by 30.1 hours (from 40.7 hours [95% CI 24.8–48.0] to 10.6 hours [95% CI 7.7–16.3], p<0.001). The results of the log-rank tests confirmed the presence of significant differences between the two periods in both steps (p<0.001).

### Time to optimal therapy

**Fig 3:**
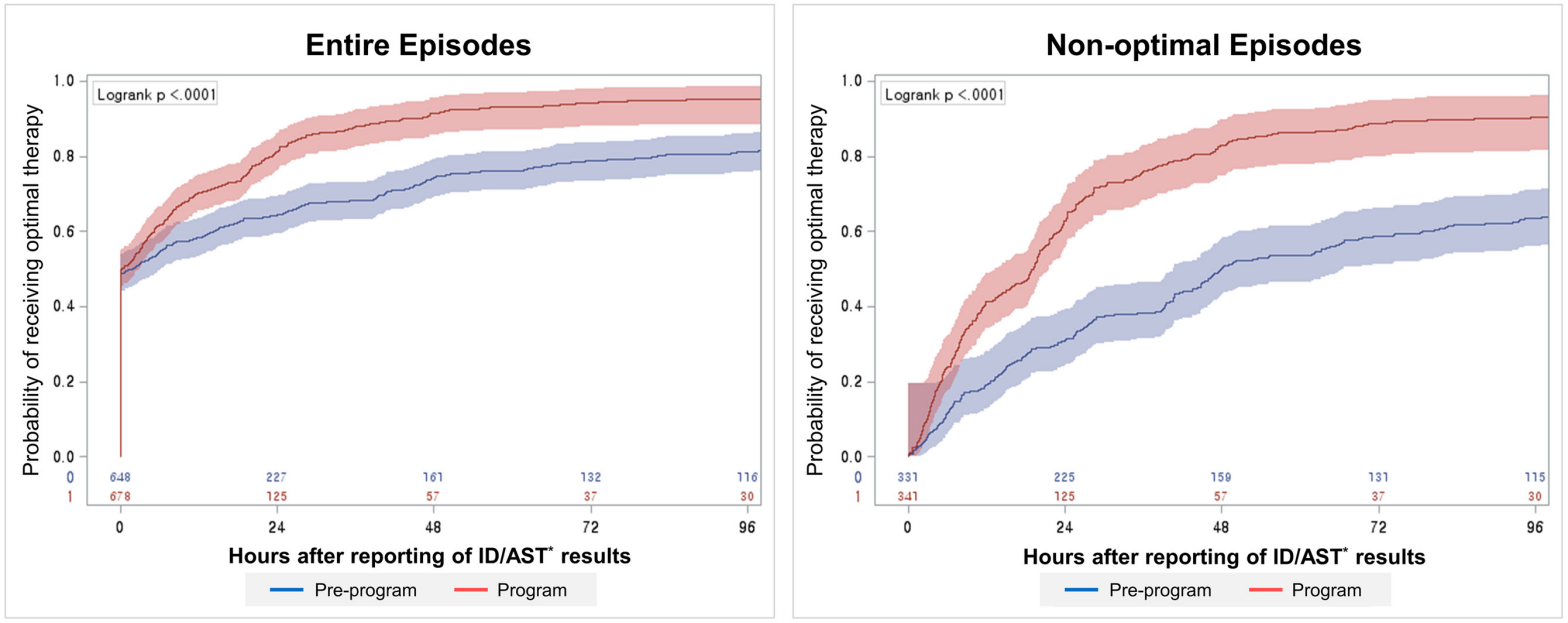
Kaplan-Meier curve of the time to optimal therapy. *Identification and Antimicrobial susceptibility test.

The proportions of optimal therapy at 24 and 48 hours increased after the reporting of the ID/AST results from 64.4% (95% CI 60.8–68.1) and 74.3% (95% CI 70.9–77.6) in the pre-program period to 81.4% (95% CI 78.4–84.3) and 91.4% (95% CI 89.3–93.5), respectively, in the program period for all episodes (Fig 3). For non-optimal episodes, the proportion of cases that received optimal therapy at 24 and 48 hours increased from 31.0% (95% CI 26.0–36.0) and 50.2% (95% CI 44.8–55.6) to 63.0% (95% CI 57.8–68.1) and 83.0% (95% CI 78.9–87.0), respectively. The median time to optimal therapy was shortened in the program period by 27.5 hours (from 46.6 hours [95% CI 40.8–55.2] to 19.1 hours [95% CI 15.4–20.6], p<0.001) (Fig 3). The results of the log-rank test revealed the presence of significant differences between the two periods in both steps (p<0.001).

### Time to de-escalation therapy

**Fig 4:**
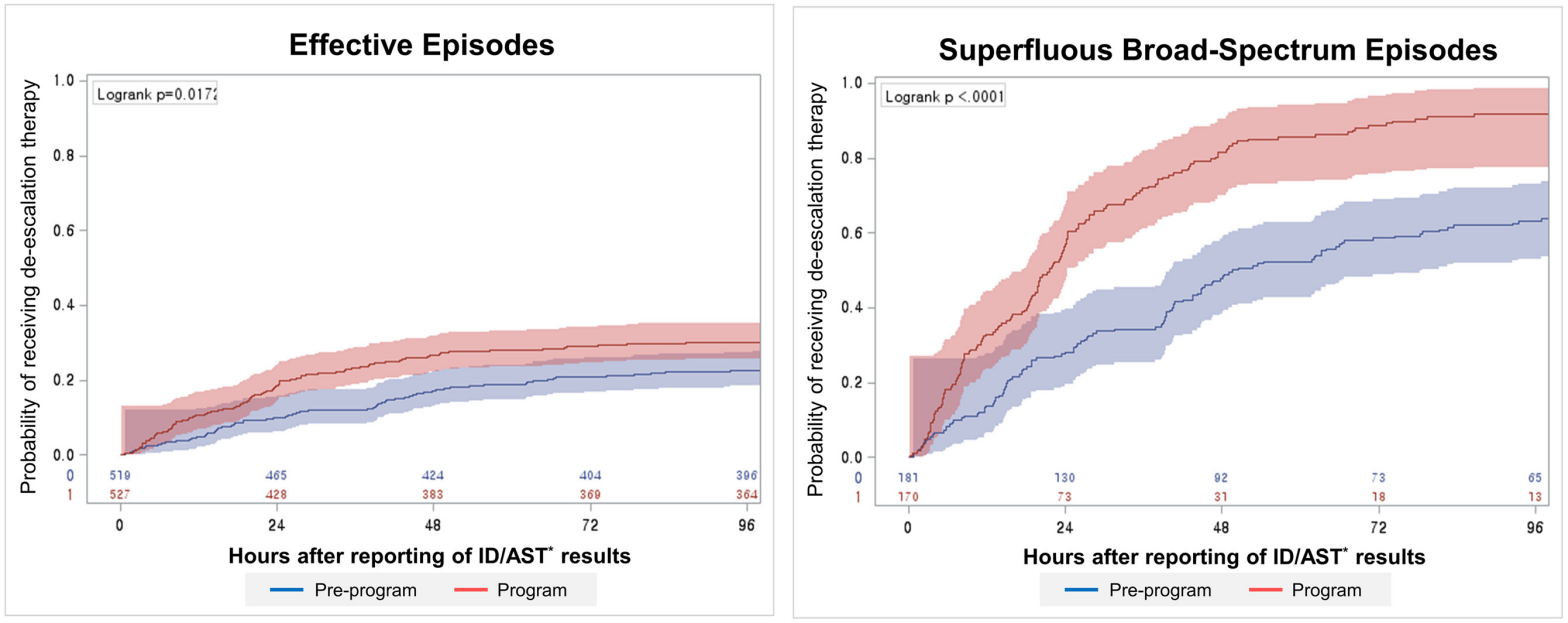
Kaplan-Meier curve of the time to de-escalation therapy *Identification and Antimicrobial susceptibility test.

Among the effective episodes, the proportions of de-escalation therapy at 24 and 48 hours after the reporting of the ID/AST results increased from 10.0% (95% CI 7.5–12.6) and 17.0% (95% CI 13.8–20.3) in the pre-program period to 18.6% (95% CI 15.3–21.9) and 26.6% (95% CI 22.8–30.4) in the program period, respectively (Fig 4). Among the superfluous broad-spectrum episodes, the proportion of de-escalation therapy at 24 and 48 hours increased from 28.2% (95% CI 21.6–34.7) and 47.8% (95% CI 40.5–55.1) to 57.1% (95% CI 49.6–64.5) and 81.5% (95% CI 75.6–87.3), respectively. The median time to de-escalation therapy was shortened in the program period by 27.9 hours (from 48.3 hours [95% CI 42.0–65.9] to 21.6 hours [95% CI 18.8–24.2], p<0.001) (Fig 4). The results of the log-rank test revealed the presence of significant differences between the two periods in both steps (p = 0.017 and <0.001, respectively).

### Time to intravenous to oral switch

The proportion of switch therapies performed on days 2, 4, and 8 increased from 8.5% (95% CI 6.4–10.7), 14.1% (95% CI 11.4–16.8), and 22.2% (95% CI 18.9–25.4), respectively, in the pre-program period to 9.1% (95% CI 7.0–11.4), 16.1% (95% CI 13.3–18.9), and 24.4% (95% CI 21.1–27.7), respectively, in the program period. However, Kaplan-Meier analysis and the log-rank test showed that there was no statistically significant difference between the times to intravenous to oral switch in the two periods (p = 0.447).

### Results of segmented linear regression analysis

The results of segmented linear regression analyses are shown in Table 3. The monthly proportion of patients on effective, optimal, and de-escalation therapies at 24 and 48 hours from the reference time increased immediately after the implementation of the program. There were no significant baseline trends and no significant changes in trends after the implementation of the program. The Durbin-Watson statistic revealed no significant autocorrelation.

**Table 3:** Parameter estimates and *p*-values from the segmented linear regression analysis: proportion of effective, optimal, de-escalation, and intravenous to oral switch therapies per month.

	Intercept		Baseline trend		Change in level		Change in trend	
	Coefficient	*p*-value	Coefficient	*p*-value	Coefficient	*p*-value	Coefficient	*p*-value
Effective at 24 hours	0.8794	<0.001	0.0003	0.934	0.0824	0.015	-0.0034	0.451
Optimal at 24 hours	0.6595	<0.001	-0.0020	0.699	0.2458	<0.001	-0.0072	0.333
De-escalation at 24 hours	0.1165	<0.001	-0.0026	0.532	0.1118	0.010	0.0013	0.823
Intravenous to oral switch at 24 hours	0.0539	0.002	-0.0010	0.620	-0.0055	0.781	0.0034	0.246
Effective at 48 hours	0.9267	<0.001	0.0009	0.745	0.0548	0.067	-0.0025	0.553
Optimal at 48 hours	0.7633	<0.001	-0.0014	0.726	0.1967	<0.001	-0.0025	0.649
De-escalation at 48 hours	0.1759	<0.001	0.0007	0.868	0.0842	0.049	-0.0011	0.853
Intravenous to oral switch at 48 hours	0.0987	<0.001	-0.0007	0.821	-0.0407	0.165	0.0063	0.135

### Length of stay and mortality rate

The number of episodes in which the outcome of the admission treatment was death was 85 in the pre-program period and 89 in the program period. These cases were excluded from the estimation of length of stay. There was no significant difference in the median length of stay between the two periods (16.6 [interquartile range (IQR) 8.8–29.8] versus 16.0 [IQR 9.5–28.9], p = 0.715). There was also no significant difference in all-cause 30-day mortality rate between the two periods (68/648 versus 67/678, p = 0.713). The infection-related 30-day mortality rate decreased (39/648 versus 27/678), but there was no statistical significance (p = 0.089).

### Subjects in the program period whose consultations were canceled

When we compared the 22.0% (149/678) whose automatically generated consultations were canceled to the remaining 78.0% (529/678), log-rank tests revealed there were no significant differences in time to effective, optimal, de-escalation, and intravenous to oral switch therapies (S1 Table). Length of stay (16.4 [IQR 8.9–35.3] versus 16.0 [IQR 9.7–27.2], p = 0.500) and 30-day mortality rate (54/475 versus 13/136, p = 0.592) were not significantly different between the two groups.

However, the proportion of subjects on effective (87.2% [130/149] versus 75.0% [397/529], p = 0.002) and optimal (60.4% [90/149] versus 46.7% [247/529], p = 0.003) antimicrobials at the reference time was higher in the canceled subgroup. The proportion of subjects on superfluous broad-spectrum antimicrobials at the reference time was not significantly different (24.8% [37/149] versus 25.1% [133/529], p = 0.939). The proportion of cases in which infectious diseases consultations were issued between 24 hours before the BSI onset and the reference time was higher in the canceled subgroup (51.0% [76/149] versus 45.0% [238/529]) without statistical significance (p = 0.193).

## Discussion

This study showed that the intervention program in our institution promoted appropriate antimicrobial prescription in the treatment of BSI. Kaplan-Meier analyses and log-rank tests revealed that the probabilities of receiving effective, optimal, and de-escalation therapies were higher in the program period. Furthermore, time to effective, optimal, and de-escalation therapies was shortened in the program period. Segmented linear regression analysis revealed that the proportion of effective, optimal, and de-escalation therapies increased after the implementation of the program. No significant trends or changes in trends were identified before or after the implementation of the program. We did not detect improvements in time to intravenous to oral switch, reduction in length of stay and 30-day mortality rate.

The program consisted of alerts and automated consultations. Alerts coupled with antimicrobial stewardship intervention [15], and routine consultations [16,17] have been proposed as effective forms of antimicrobial stewardship program (ASP) for limited subjects. Our study included a complete set of BSIs and involved a novel form of stewardship program which could improve the appropriateness of antimicrobial prescription.

Intervention by IDS is a key element in optimizing antimicrobial therapy, and previous studies have shown that IDS services and consultation improved antimicrobial prescription and clinical outcomes [6,8,9,18]. Our program was also based on interventions conducted by IDS. However, automatically generated consultations were canceled in 22.0% of the subjects in the program period. We could not evaluate the reasons for cancelation and it was impossible to clearly separate the effects of electronic alerts and automated consultations. The cancelation could be related to the consultation to IDS before the reporting of the ID/AST results. However, there was no statistically significant difference in the proportion of consultation to IDS between the canceled 22% and the remaining 78%.

Another remarkable feature of our program was the use of information technology resources. Interventions based on electronic health records (EHRs) and clinical decision support systems (CDSSs) have been predicted to increase the efficiency of ASP [19,20]. In our study, the electronic alerts may have had a synergistic effect in improving antimicrobial prescription, and the use of electronic medical records may have enabled faster and more accurate communication. The mean time to signed replies in the automated consultations of only 6.8 hours (95% CI, 5.8 to 7.8) achieved here might not have been possible by human effort alone.

There has not been any unified definition of appropriate antimicrobial use [1,8]. Other terms such as “adequate use” or “optimal use” have been employed with similar meaning. Usually these words were applied only to activity in vitro. Interventions to improve antimicrobial use should not just aim for early initiation of active antimicrobial therapy. By developing comprehensive definitions, we aimed to simultaneously initiate active antimicrobial therapy and narrow the coverage. We have shown that our program achieved both aims. Furthermore, the impact of our program in avoiding unnecessary antimicrobial use might be underestimated because we did not include the cases regarded as contaminants in the analyses.

However, our approach had no significant impact on the intravenous to oral switch. We suspected that this was due to the relatively low cost of hospitalization and the lack of economic incentives to reduce the duration of hospitalization in our health care system. This reasoning could also be applied to the issue of length of stay. With respect to the extended average length of stay in hospitals, the Republic of Korea had ranked the second among OECD member countries recently [21]. Since a considerable proportion of the total episodes (40.4%) were categorized as hospital-onset, the program’s impact on length of stay could not be substantial.

In the program period, the interval from the BSI onset to the reporting of the ID/AST results decreased. We assumed that this finding was the result of placing dedicated staffs in our laboratory since June 2011. Although the decrease in the reporting time was not directly related to the program, it might influence the overall time to appropriate antimicrobial therapy. Despite this subsidiary change and the promotion of appropriate antimicrobial prescriptions, there was no significant improvement in the 30-day mortality rate after the initiation of the program. The possibility of improvement in the mortality rate might be small because approximately 80% of the study subjects were already on effective antimicrobials at the reference time. In addition, the impact of our intervention on the mortality rate might be limited owing to the considerable portion of older individuals with a high comorbidity index. First, it is possible to infer that the critical time point may be some time before the reference time of our study [9,22]. Nonetheless, considering that our program promoted the administration of effective antimicrobials and de-escalation therapy concurrently, both the absence of an increase in all-cause mortality rate and the tendency of improvement in the infection-related mortality rate can be considered meaningful. Mandatory consultations, as in the cases of *Staphylococcus aureus* BSI [16,17], may enhance the impact of our program on clinical outcomes.

Despite its limitations, the combination of electronic alerts and automated consultations aimed at BSI treatment was found to improve antimicrobial prescription in the current study. Our approach offers an efficient form of stewardship intervention and demonstrates the possibility of exploiting informatics. However, additional efforts to identify more effective and efficient forms of stewardship program using information technology are needed. Also studies to establish the most appropriate time for intervention in terms of efficiency and practicality are warranted. The point in time when the results of the Gram stain are reported may represent the appropriate time for an intervention. However, an earlier intervention will require more resources and intensive work. The introduction of novel diagnostic tools such as matrix-assisted laser desorption/ionization time-of-flight mass spectrometry (MALDI-TOF MS) and *S*. *aureus* PCR [23,24] offer further opportunities for stewardship programs for BSI.

## Supporting Information

S1 Fig Schematic diagram of the time course of the program. ^a^Emergency department. ^b^Identification and Antimicrobial susceptibility test.
(TIF)

S2 Fig A flow chart of describing selection of the subjects included in the analysis. (TIF)

S3 Fig Classification of empirical therapy. (TIF)

S1 Table Log-rank *p*-values according to the subgroups. (PDF)
