# Supplementary figures and images for: Electronic Alerts with Automated Consultations Promote Appropriate Antimicrobial Prescriptions
Source: PLoS One. 2016 Aug 17;11(8):e0160551. doi: 10.1371/journal.pone.0160551 (PMC4988717; doi:10.1371/journal.pone.0160551)

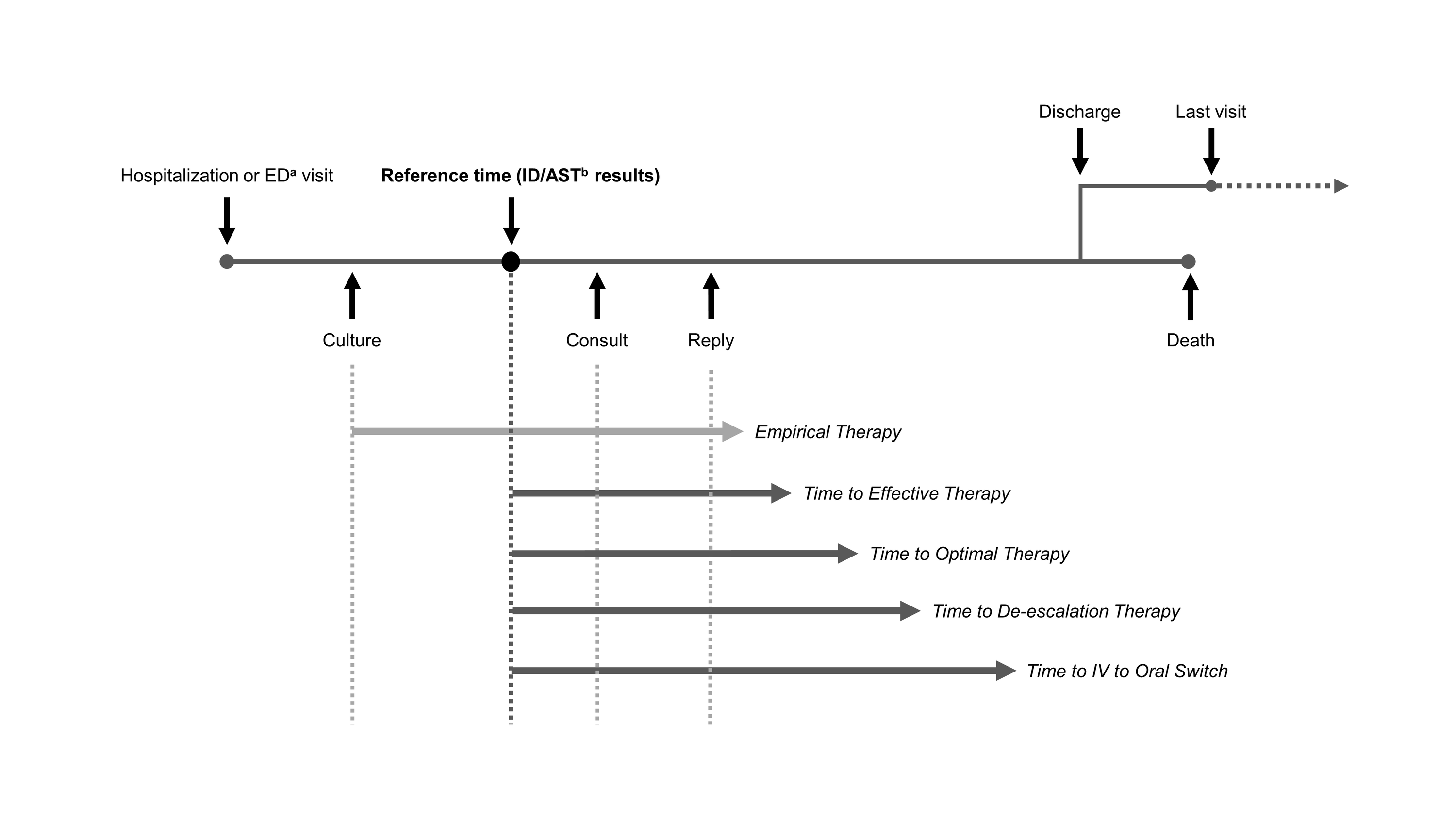

Supplement: S1 Fig — aEmergency department. bIdentification and Antimicrobial susceptibility test. (TIF) [file pone.0160551.s001.tif]

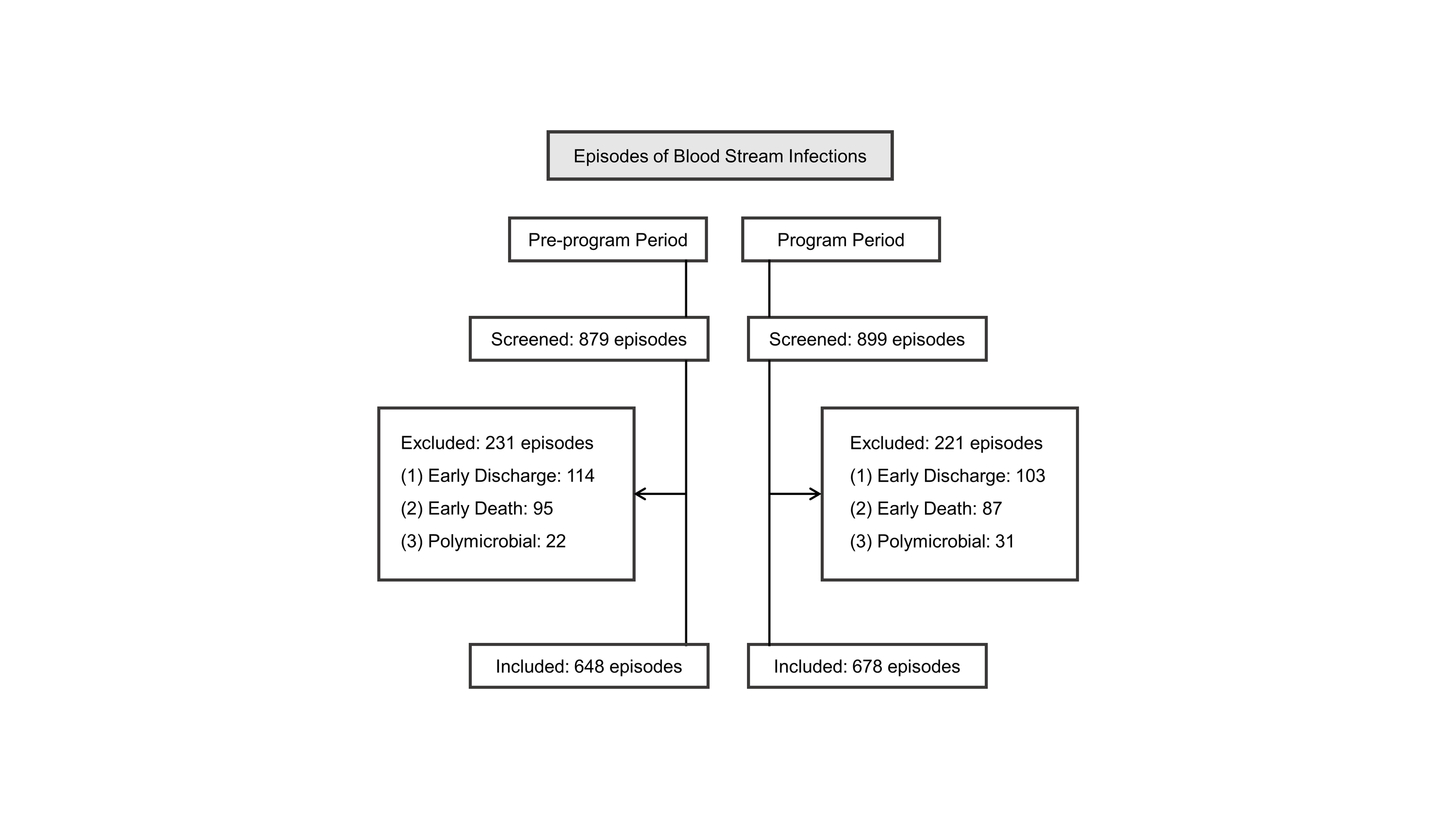

Supplement: S2 Fig — (TIF) [file pone.0160551.s002.tif]

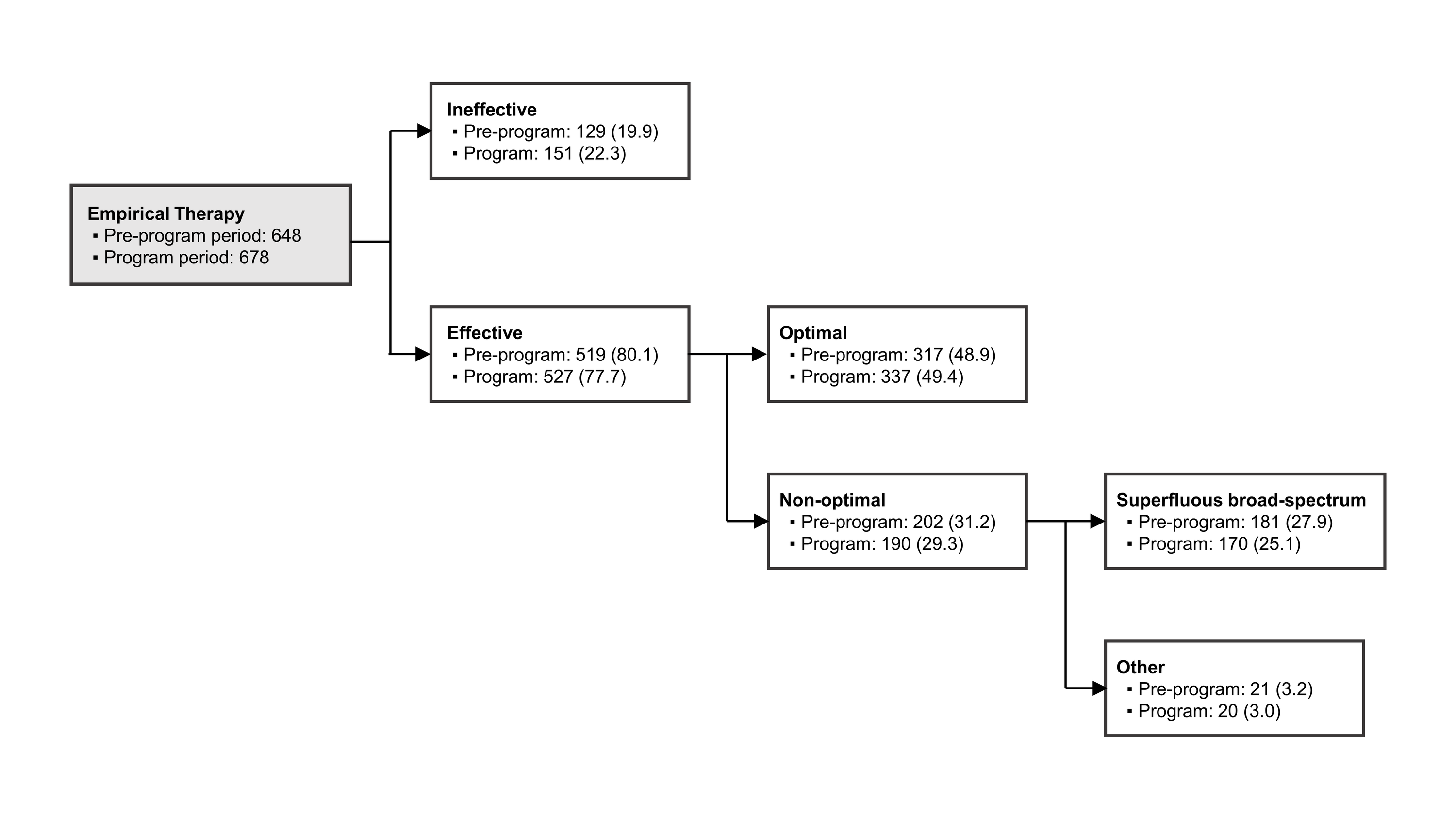

Supplement: S3 Fig — (TIF) [file pone.0160551.s003.tif]
