# Supplementary material for: Electronic Alerts with Automated Consultations Promote Appropriate Antimicrobial Prescriptions
Source: PLoS One. 2016 Aug 17;11(8):e0160551. doi: 10.1371/journal.pone.0160551 (PMC4988717; doi:10.1371/journal.pone.0160551)
Supplement: S1 Table — (PDF) [file pone.0160551.s004.pdf]

S1 Table. Log-rank *p*-values according to the subgroups

	Time to effective	Time to optimal	Time to de-escalation	Time to intravenous to oral switch
22% vs. 78%*	0.075	0.345	0.872	0.810
Pre-program vs. 22%	0.002	<0.001	0.088	0.233
Pre-program vs. 78%	<0.001	<0.001	0.014	0.126

*22%: canceled subgroup, 78%: not canceled subgroup
